# Supplementary figures and images for: Development of a Model System to Identify Differences in Spring and Winter Oat
Source: PLoS One. 2012 Jan 9;7(1):e29792. doi: 10.1371/journal.pone.0029792 (PMC3253801; doi:10.1371/journal.pone.0029792)

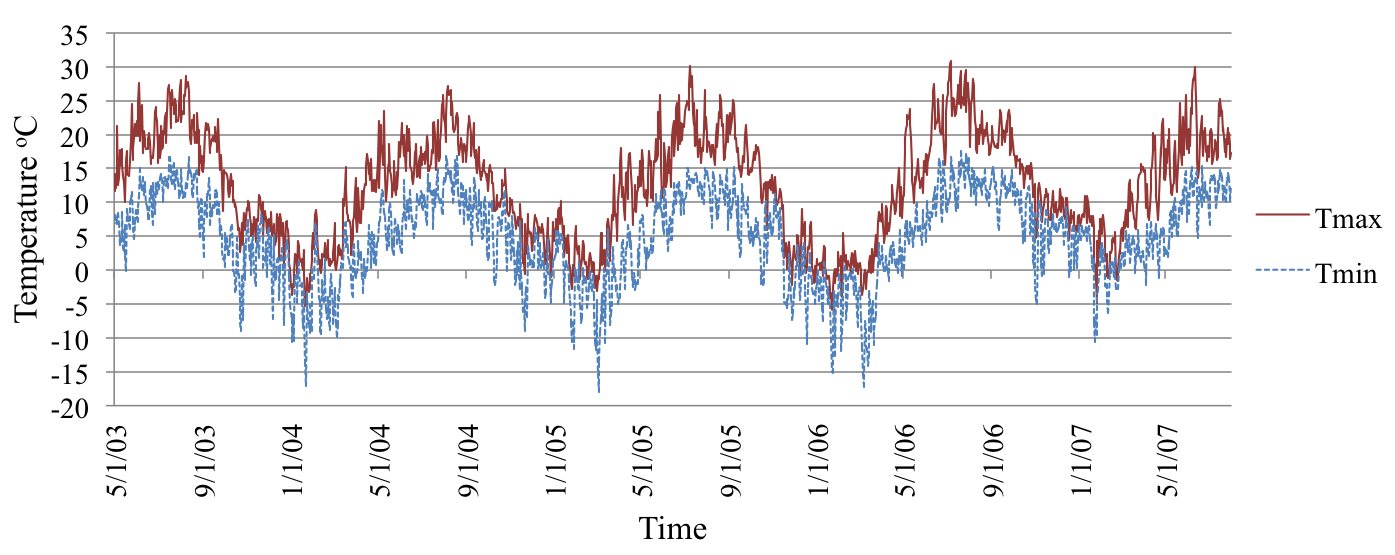

Supplement: Figure S1 — Temperature data from the Svalöv area from 2003–2007. Daily maximum and minimum temperatures from May 2003 till July 2007 for the fields in Svalöv obtained by Mesoscale analysis system from SMHI. (TIF) [file pone.0029792.s001.tif]

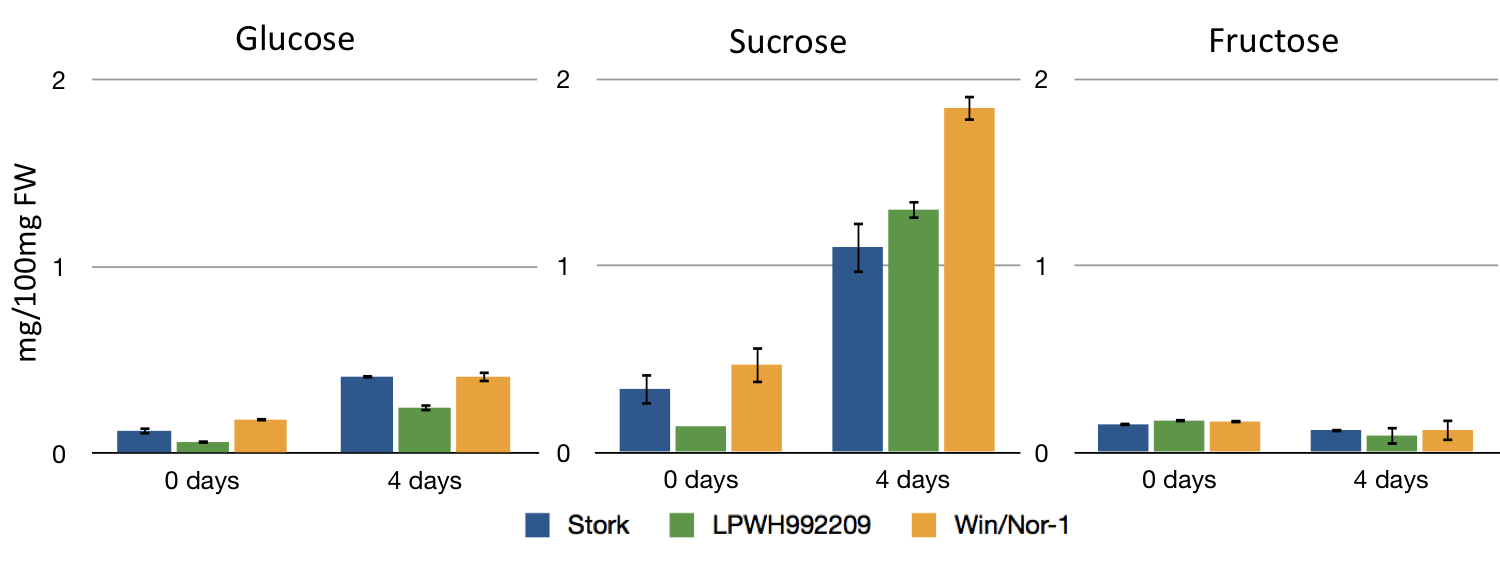

Supplement: Figure S2 — Sugar analysis by enzymatic assay. Sugar analysis (glucose, sucrose or fructose) in Stork (blue), LPHW992209 (green) and Win/Nor-1 (orange). Pooled leaf samples were collected from two weeks old non-acclimated plants (0 days) and cold acclimated plants (2 and 4 days). Approximately 0.5 g of leaf tissue was lyophilised and incubated for 10 min in 2 ml 80% ethanol in a water bath set at 90°C. The extract was then filtered through 20 µm nylon mesh. The incubation and filtration was repeated once more on the leaf tissue. Finally, the extract was centrifuged for 5 min at 4000 g. Glucose concentration was determined by Glucose (HK) assay kit (GAHK-20, Sigma Aldrich), sucrose by Sucrose assay kit (SCA-20, Sigma Aldrich) and fructose by fructose assay kit (FA-20, Sigma Aldrich). The samples were analysed within 6 hours of extraction. Error bars are SEM. (TIF) [file pone.0029792.s002.tif]
